# Supplementary material for: Application of the Social Cognitive Theory amid food parenting practices of Black immigrant mothers in the US: A qualitative study
Source: PLoS One. 2024 Aug 8;19(8):e0298847. doi: 10.1371/journal.pone.0298847 (PMC11309485; doi:10.1371/journal.pone.0298847)
Supplement: Attachment — Submitted filename: Response to Reviewers.docx [file pone.0298847.s001.docx]

June 28, 2024

Dear Editor,

The authors would like to thank the reviewers for their fair reviews and detailed list of constructive suggestions. We appreciate the opportunity to revise our manuscript for consideration of publication in the PLOS ONE journal. We have responded to each comment from the editors as well as the reviewers. Point-by-point Responses are given below (page 2 - 12) in italics.

We believe that the manuscript is now suitable for publication in PLOS ONE.

Regards,

Phoebe Tchoua, DrPH, MPH

On behalf of all authors

Reviewer 1

1. Please ensure that your manuscript meets PLOS ONE's style requirements, including those for file naming. The PLOS ONE style templates can be found at

https://journals.plos.org/plosone/s/file?id=wjVg/PLOSOne_formatting_sample_main_body.pdf and

<https://journals.plos.org/plosone/s/file?id=ba62/PLOSOne_formatting_sample_title_authors_affiliations.pdf>

***RESPONSE****: The manuscript has been modified to meet the PLOS ONE style requirements.*

2. Thank you for stating the following financial disclosure: "This research was supported by a grant from the T32 Cancer Health Disparities Training Grant from the National Cancer Institute of the National Institutes of Health (T32CA128582). "

Please state what role the funders took in the study. If the funders had no role, please state: ""The funders had no role in study design, data collection and analysis, decision to publish, or preparation of the manuscript.""

If this statement is not correct you must amend it as needed.

Please include this amended Role of Funder statement in your cover letter; we will change the online submission form on your behalf.

***RESPONSE****: The funder had no role in the preparation of the manuscript. The edited version of the financial disclosure now reads:* "The funders had no role in study design, data collection and analysis, or preparation of the manuscript."

3. Please include your full ethics statement in the ‘Methods’ section of your manuscript file. In your statement, please include the full name of the IRB or ethics committee who approved or waived your study, as well as whether or not you obtained informed written or verbal consent. If consent was waived for your study, please include this information in your statement as well.

***RESPONSE****: The ethics statement has been modified as requested. Please see lines* 118-121: ” The study protocol was reviewed and approved by the East Tennessee State University (ETSU) Institutional Review Board. Digital consent, by clicking “I agree: (a) I have read the consent form, (b) I agree to volunteer, and (c) I am physically present in the United States”, was gained from each participant prior to study involvement.”

Reviewer's Responses to Questions

Comments to the Author

1. Is the manuscript technically sound, and do the data support the conclusions?

The manuscript must describe a technically sound piece of scientific research with data that supports the conclusions. Experiments must have been conducted rigorously, with appropriate controls, replication, and sample sizes. The conclusions must be drawn appropriately based on the data presented.

Reviewer #1: Partly

Reviewer #2: Partly

2. Has the statistical analysis been performed appropriately and rigorously?

Reviewer #1: I Don't Know

Reviewer #2: Yes

3. Have the authors made all data underlying the findings in their manuscript fully available?

The PLOS Data policy requires authors to make all data underlying the findings described in their manuscript fully available without restriction, with rare exception (please refer to the Data Availability Statement in the manuscript PDF file). The data should be provided as part of the manuscript or its supporting information, or deposited to a public repository. For example, in addition to summary statistics, the data points behind means, medians and variance measures should be available. If there are restrictions on publicly sharing data—e.g. participant privacy or use of data from a third party—those must be specified.

Reviewer #1: Yes

Reviewer #2: Yes

4. Is the manuscript presented in an intelligible fashion and written in standard English?

PLOS ONE does not copyedit accepted manuscripts, so the language in submitted articles must be clear, correct, and unambiguous. Any typographical or grammatical errors should be corrected at revision, so please note any specific errors here.

Reviewer #1: Yes

Reviewer #2: Yes

5. Review Comments to the Author

Please use the space provided to explain your answers to the questions above. You may also include additional comments for the author, including concerns about dual publication, research ethics, or publication ethics. (Please upload your review as an attachment if it exceeds 20,000 characters)

**Reviewer #1: TO THE AUTHORS**

SUMMARY This study evaluated Social Cognitive Theory constructs, personal (maternal knowledge, attitudes, and beliefs), and environmental factors (acculturation) in relation to food parenting practices in Black immigrant mothers residing in the US. Data analysis revealed that knowledge, attitude, belief, modeling, acculturation, coercive control, and structure, influenced food parenting practices and their children’s eating habits. Children consumed both the mother’s native diet and the American diet. Mothers encouraged healthy foods by eating more fruits and limiting unhealthy foods for their children, although children snacked more due to work schedules, food costs and accessibility. In conclusion, this study identified the food parenting practices of Black immigrant women associated with the dietary habits of their children.

Indeed, there is a gap in the literature of studies focusing on the dietary behavior of migrant families residing in the US. Given that the US is a multicultural society, from a public health perspective, this topic would be of interest to health professionals working with immigrants in the US. Overall, a good effort was made by the authors. However, there are areas that require clarification that will improve study comprehensibility, transparency and replication. Please refer to my comments to the authors

COMMENTS TO THE AUTHORS

MAIN MANUSCRIPT

**Introduction**

1. -line 83, add references to the few studies

***RESPONSE****: Thank you for pointing it out. We did not find any study in the US for Black immigrant mothers. We deleted the first sentence (line 82)*

**Methods**

2. -Line 101, 'All participants providThe first focus group included mothers of children aged 2-4 years old.

Revise is not comprehensible.

***RESPONSE****: Thank you for pointing it out. We revised the text. Deleted “*'All participants provid*” (line102)*

3. Line 103, 'Thirty mothers from seven African and Caribbean countries participated in the four focus group discussions. ' List the 7 countries.

***RESPONSE****: Thank you for pointing it out. We added the name of the 7 countries. (Line104-105)*

Line 104 – 106: “Thirty mothers from seven African and Caribbean countries, Cameroon, Ghana, Kenya, Nigeria, South Africa, Senegal, Trinidad and Tobago, participated in the four focus group discussions.”

4. Lines 121-127 can be deleted 'All four focus groups were moderated by PT...............,The research team (PT, SZ, and SM) facilitated the sessions.

***RESPONSE****: We deleted those lines. (lines 123-130)*

5. In the methods section, assessment tools should be described, namely the questionnaire. The role and contribution of each co-author should be mentioned in the Author's contribution section. Any researchers who assisted in data collection and who are not co-authors should be mentioned in the acknowledgement section of the paper (i.e., SZ, SM).

***RESPONSE****: The measure section includes the type of questionnaire used and it is also in Table 1. We removed the research assistant initials.*

6. Was this questionnaire validated in a pilot study and tested for comprehensibility?

If yes, add a reference to this study or a protocol study. If not, mention this in the study limitations.

***RESPONSE****: Thank you for pointing it out. No, the questionnaire was not validated in a pilot study. It was reviewed after each focus group. We added this as a limitation in the limitation section (line 689-690)*

*Line 689-690: “*First, the interview guide had not been previously validated in a pilot study and tested for comprehensibility.*”*

Section 2.3 Measures should be revised.

7. Line 137 'The hypothesis was specified before the data were collected'.

State the hypothesis under investigation on line 94 of the introduction, and delete line 137 from Section 2.4 Data Collection.

***RESPONSE****: Thank you for pointing it out. We added a sentence in the introduction section (line 94-95) about the hypothesis. “This study was exploratory; therefore, the researchers did not have any prior hypotheses.”*

*Hypothesis information in section 2.4 was deleted (line 140)*

8. Lines 143-153 ‘A codebook was generated through an iterative process whereby themes were generated from the focus group data. Inter-rater reliability was assessed through a process where 25% of the data were double-coded by PT and MB (the second author, and a doctoral student experienced in qualitative data ……….following completion of the research project.’

Line 146 delete author initials. 'by PT and MB (the second author, and a doctoral student experienced in qualitative data analysis'

***RESPONSE****: We deleted the initials. (lines 161, 165, 1667)*

9. line 148-149, Differences in codes were discussed, and resolved, and the codebook was modified accordingly to help with consensus building and inter-rater reliability.

Was a statistical test used to assess inter-rater reliability, e.g. Cohen's Kappa?

***RESPONSE***: Thank you for pointing it out. *We used percent agreement.*

Line 155-157: “Inter-rater reliability was assessed through a process where 25% of the data [21] were double-coded using percent agreement among raters as described in McHugh 2012 [22].”

10. Line 150 ‘reached 83% coding similarity;

How was 83% coding similarity calculated? Mention this in text.

***RESPONSE***: *We counted the number of similar codes and divided that number by the total number of codes to obtain the interrater reliability percent. The text was edited to clearly state how we calculated the coding similarity.*

Line 155-166: “Inter-rater reliability was assessed through a process where 25% of the data [[21]](https://sciwheel.com/work/citation?ids=10576420&pre=&suf=&sa=0&dbf=0) were double-coded using percent agreement among raters as described in McHugh 2012 [[22]](https://sciwheel.com/work/citation?ids=2160610&pre=&suf=&sa=0&dbf=0). Each rater coded the same data independently using a predetermined unique color for each code. Next, one rater counted the number of similar codes and divided that number by the total number of codes to obtain the interrater reliability percent. The initial percentage was less than 80 and both raters met to discuss and resolve discrepancies. Following the discussions, the codebook was modified accordingly to help with consensus building and inter-rater reliability, and percent agreement between raters increased. The team reached 83% coding similarity;”

**Results**

11. Line 155, ‘During two weeks in August 2022.’ Redundant, delete from text.

Study duration was mentioned on line 98 of methods.

***RESPONSE****: Thank you for pointing it out. We deleted that part of the text. (line 171)*

12. Lines 157-159 ‘The focus groups were held on Tuesday evenings and Saturday mornings or early afternoon. Seven countries were represented across all focus groups, with most participants emigrating from African countries’.

Move to methods under the subheading of data collection/data analysis.

***RESPONSE****: Thank you for pointing it out. We moved lines 173-1175 to line 146-148.*

14. Line 183, delete 'body-building foods' not appropriate word, replace by nutritious foods

***RESPONSE***: *‘body-building’ was a word used by a study participant. The word has been replaced with ‘nutritious foods’ as suggested.*

Line 199: “On the other hand, participants shared that eating nutritious foods such as fruits and …”

15. Line 223, define in brackets 'fufu' (legume?)

***RESPONSE***: *Thank you for pointing it out*. *We added a definition in brackets.*

Line 239: ““*Growing up I hated beans and fufu [pounded meal of starchy root vegetables or corn].*”

**Discussion**

16. lines 564-565 'This finding agrees with [21] results, nutritional belief was significantly associated with feeding style. Also, mothers confidently shared which foods the child should eat and why’

Ambiguous. Elaborate by outlining the study by Boucher et al (ref 21)

***RESPONSE***: *Thank you for pointing it out*. *We* *deleted that sentence. (Lines 581-582)*

17. Line 573-576 , 'On the other hand, participants also shared what additional information they would like to ............to consume from each food group.’

Are you referring to the present study or ref 21? It is not clear from this statement. Revise

***RESPONSE***: *We are referring to the present study. We added “…focus group participants to make that clearer”*

Line 590: “On the other hand, focus group participants also shared what additional information they…”

18. Line 588 'As previously SCT has been used to help explain human behaviour'.

Add a reference to support this statement.

***RESPONSE***: *We added 2 references.*

Line 606: “As previously mentioned, SCT has been used to help explain human health behavior [[16, 26]](https://sciwheel.com/work/citation?ids=15860782,1943147&pre=&pre=&suf=&suf=&sa=0,0&dbf=0&dbf=0).”

19. Line 677. ‘Another strength of this study is the 83% inter-rater reliability in data coding which reduced qualitative study bias.’

In data-analysis, mention which statistical test was used to derive 83% inter-rater reliability.

***RESPONSE***: We a*dded the method used to reach 83% inter-rater reliability, “percent agreement”.*

Lines 696-697: “Another strength of this study is the 83% inter-rater reliability, through percent agreement among raters, in data coding which reduced qualitative study bias.”

20. Line 682 ‘We minimized this risk by establishing ground rules at the beginning of each focus group’

What do you mean by ground rules? Inclusion criteria?

***RESPONSE***: *Thank you for pointing it out. We added examples of the ground rules in parentheses to make that statement clearer.*

Lines 703 – 705: “We minimized this risk by establishing several ground rules (e.g., identities of group members will remain confidential; group members have a right to their viewpoints and opinions) at the beginning of each focus group.”

**Conclusion**

The conclusion should concisely summarize the main findings.

21. line 689-691, ‘To the authors’ knowledge, this is the first study in the US to explore the food parenting practices of Black immigrant mothers while informed by the SCT’

Delete. This statement was mentioned on line 670 of the discussion

***RESPONSE***: *We* *deleted that statement. (Lines 711-713)*

22. Line 695 ’The child’s dietary behavior, food choice, and eating behavior can be predictive of adiposity and the attendant morbidity that accompanies excess body weight [36, 37].’

Delete this statement. High BMI was not evaluated in this study.

***RESPONSE***: *Thank you for pointing it out. We deleted the entire sentence (Lines 717 – 718)*

23. Line 697 ‘This study highlights essential areas to consider when developing nutritional or obesity interventions for this population.’

Revise, for the same reason as above.

***RESPONSE***: *We deleted the word ‘obesity’. (Line 719)*

Lines 718-7120: “This study highlights essential areas to consider when developing nutritional or diet quality interventions for this population.”

**References**

24. Ref 7 UNICEF. Add link to the website.

***RESPONSE***: *Thank you for pointing it out.* We *added link to website*.

 [7] U.N.I.C.E.F. Early Childhood Nutrition, https://www.unicef.org/nutrition/early-childhood-nutrition (accessed May 27, 2024).

**Reviewer #2**: This is an excellent and well-written paper that explored a well-known and topical issue but in an uncharted subpopulation in the US. The study aimed to use the Social Cognitive Theory (SCT) framework to assess the constructs of maternal knowledge, attitudes, beliefs, acculturation, and modeling in relation to food parenting practices among a sample of Black immigrant mothers living in Metro Atlanta, Georgia.

The sought to Identify and explore these defined SCT constructs as key factors that influence the food parenting practices (FPP) of this population in relation to their children’s dietary habits.

1. In the abstract, the author’' conclusion implied that the study identified“"key factors that influence food parenting practice”" and as well“"childre’'s dietary habit”". However the lowest end in the pathway of their SCT construct stopped at FPP. This calls for a delicate balancing of what could or not be concluded by this study.

***RESPONSE***: *Thank you for pointing it out. “Obesity prevention” was removed from the abstract conclusion (line 45).*

2. The authors should reconsider their statement under data analysis that "hypothesis was specified" suggesting that the attempt was to test a hypothesis, which is outside the remit of qualitative research. Qualitative research 'explains' the how or why a phenomenon occurs, and not necessary 'establish' relationship.

***RESPONSE***: *Thank you for pointing it out. We deleted Line 140: “The hypothesis was specified before the data were collected”*

3. The authors did very well in organizing the 'predefined' and emergent themes and subthemes. However, one could only imply that perhaps both closed and open coding approaches were employed; this should be explicitly stated. That way, it would be clear that some subthemes, for instance, "know" which apparently has the same presumed definition as the theme "knowledge", had been predefined, and is clearly one of the preset SCT constructs that the study aimed to assess.

***RESPONSE***: *Thank you for pointing it out*. *The description of the analysis on Lines 142-144 has been expanded to describe the inclusion of the SCT constructs as well as the themes that emerged during the open analysis of the data*.

Lines 141-146: “The data analysis was thematic [[20]](https://sciwheel.com/work/citation?ids=901185&pre=&suf=&sa=0&dbf=0) following the modified SCT constructs and some questions from the focus group semi-structured discussion guide as central themes. Analysis also allowed for the identification of emergent themes beyond those contained within the SCT model. The researchers combined inductive and deductive coding approaches.”

4. The authors could also consider a further analysis of the theme, "attitude", as it appears that there are subthemes that may bother on the presence versus absence of self-conflicts. There is a suggestion that some of the mothers themselves may be conflicted in what emerged as the attitudes that they displayed.

***RESPONSE***: *In considering the utility of further analysis of the attitude theme, it may be helpful to note that the construct of self-conflict is included as a part of the definition of attitude: “her feelings and emotions, her actions, and her tendency to move toward or away from certain foods.”(Lines 221-227). The inherent complexity of attitude as a construct, and the related findings presented in this manuscript, may point to a promising avenue for future research in this population. To that end, we have added a sentence to the discussion section (Lines 616-617) to point to the positive potential of additional research in this area.*

5. On a trivial note, the numbering of the themes and subthemes could be revisited; for instance, it is not clear why the first theme was numbered 3.1 and not simply 1, and the subthemes 1.1 and 1.2 in tow.

***RESPONSE***: *The numbering has been edited slightly to minimize confusion.*

6. Similarly, the Editor should advise on if or what any numbering style should feature in the Discussion section. That said, authors should note that the subheading titled, 'summary of main findings' under the Discussion, does not do justice to the depth of all that was presented here.

***RESPONSE***: *We defer to the Editor as to the needed numbering, and will update the manuscript accordingly. As to the summary of main findings section, we strove to provide a thorough yet concise summary of the novel relationships found of mothers’ culture and foodways to their parenting practices. We welcome suggestions on how we can enhance this section.*

7. In addition, authors should avoid repeating the participants' verbatim quotes (i.e., results) in the Discussion.

***RESPONSE***: *We provided summaries of comments made in the Discussion section, but no verbatim quotes. We did include one of the questions asked as a verbatim italicized phrase (Lines 619-620). We removed the question and paraphrased it.*

Lines 619-620: “When we asked mothers about their diet from their child perspective, they listed African…”

8. In the conclusion, the authors should consider revising the use of these phrases: "directly influence" and "in turn impact" given that the qualitative research approach or the findings of this study would or did not establish such level of inferences.

***RESPONSE***: *Thank you for pointing it out. We have added the word ‘may’ in both cases to avoid making such a strong statement.*

Lines 714-717: “Their personal factors (i.e., knowledge, attitude, belief) and environmental factors (i.e., acculturation) may directly influence their behavioral factors (i.e., food parenting practices) which in turn may impact their children’s dietary behavior.”

9. Also it is not typical to make conclusions by citing other researchers' work or statements [Lines 695 & 696]; given that the study did not explore child dietary behaviour or body weight, such statements could be avoided as a conclusion of this study.

***RESPONSE***: *We revised the conclusion. Deleted the sentence with references and the word ‘obesity’*

Editor should double-check lines 56, 101, and 555 for editorial issues.

*Response: We defer to the editor*.
